# Informal sale of antibiotics in Guatemalan convenience stores before and after implementation of federal antibiotic dispensing legislation

**DOI:** 10.1186/s40360-023-00720-8

**Published:** 2024-01-25

**Authors:** N. Rojop, P. Moreno, L. Grajeda, J. Romero, L. Reynoso, E. Muñoz, G. H. Palmer, C. Cordón-Rosales, D. R. Call, B. M. Ramay

**Affiliations:** 1 Department of Epidemiology, School of Sciences and Humanities, Universidad del Valle de Guatemala, Guatemala City, Guatemala; 2 Center for Health Studies, Universidad del Valle de Guatemala, Guatemala City, Guatemala; 3 Department of Pharmaceutical Chemistry, School of Sciences and Humanities, Universidad del Valle de Guatemala, Guatemala City, Guatemala; 4 Paul G. Allen School for Global Health, Washington State University, Pullman, WA United States of America

**Keywords:** Antibiotics, Prescription, Over-the-counter availability, Convenience stores

## Abstract

**Background:**

Convenience stores in Guatemala provide essential consumer goods in communities, but many dispense antibiotics illegally. Federal legislation, passed in August of 2019, requires prescriptions for antibiotic purchase at pharmacies but it is unclear if this legislation is enforced or if it has any impact on unlawful sales of antibiotics.

**Methods:**

To determine if antibiotic availability changed in convenience stores, we carried out a repeated measures study collecting antibiotic availability data before and after implementation of the dispensing regulation.

**Results:**

There was no statistical difference in the proportion of convenience stores that sold antibiotics before and after antibiotic regulations [66.6% (295/443) and 66.7% (323/484), respectively, *P*>0.96], nor in the number of stores selling amoxicillin [55.5% (246/443) and 52.3% (253/484), respectively, *P*>0.96], but fewer stores (20%) sold tetracycline capsules after regulation was passed (*P*<0.05). For stores visited both before and after passage of legislation (*n*=157), 15% stopped selling antibiotics while 25% started selling antibiotics. Antibiotics from convenience stores were reportedly sold for use in people and animals.

**Conclusions:**

Antibiotics remain widely available in convenience stores consistent with no significant change in the informal sector after implementation of prescription requirements for pharmacies. Importantly, effects from regulatory change could have been masked by potential changes in antibiotic use during the Severe Acute Respiratory Syndrome Coronavirus 2 (SARS-CoV-2) pandemic.

## Introduction

The World Health Organization (WHO) has declared that resistance to antimicrobials is one of the ten major threats to public health [1]. In 2019 there were an estimated 1.27 million deaths worldwide attributable to antimicrobial resistance [2]. The improper and excessive use of antimicrobials is one of many factors contributing the emergence of drug-resistant pathogens, and is consistent with the observation that approximately 50% of antibiotics are either prescribed, distributed, or sold improperly [3]. Antibiotics are reportedly available without prescription in 80% of countries in the Americas [4]. Furthermore, it is likely that indiscriminate use and availability of antibiotics has been exacerbated by the need for more antibiotics due to secondary bacterial infections associated with the Severe Acute Respiratory Syndrome Coronavirus 2 (SARS-CoV-2) pandemic [5].

Pharmacy establishments in Guatemala are registered and licensed to sell antibiotics, but self-medication occurs frequently and may result in inappropriate use and inability to resolve underlying pathologies. Self-medication occurs in the absence of regulations, or failure to enforce dispensing regulations, and worsens when there is insufficient oversight by trained healthcare professionals during antibiotic dispensing [6–8]. Convenience stores (*tiendas* in Spanish) dispense antibiotics, but unlike pharmacies they are not licensed to sell these products. Convenience stores provide essential consumer goods and play an important role in communities, particularly for people with limited income who purchase single unit items daily. In Guatemala, marketing studies show that 91% of the population visits convenience stores, on average, 24 times each month to purchase beverages, food, milk-products, homecare, and beauty products. Guatemalan convenience stores account for almost 50% of food sales, representing the livelihood and income for hundreds of families [9]. Antibiotics available to large volumes of customers in convenience stores may adversely impact individual health outcomes and public health.

Policies that regulate over-the-counter sales of antibiotics may have little to no impact on self-medication practices if the policies are not effectively implemented and enforced [10]. For example, a study in Saudi Arabia found that while prescription regulations impacted the attitudes of retail pharmacists about non-prescription use, there was no change in dispensing of over-the-counter (OTC) antibiotics [11]. OTC antibiotic dispensing is favored at informal establishments where attendants typically have limited knowledge about antibiotic dispensing, and they may encounter high customer demand for these products, especially in establishments where there is limited enforcement of dispensing regulations [12].

In August 2019, Guatemala approved the Ministerial Agreement 181-2019 requiring prescriptions for the dispensing of antibiotics in all private and state pharmacies [13]. This regulation was intended for establishments that are licensed to dispense antibiotics, i.e., pharmacies, but not convenience stores that supply antibiotics illegally. Given that people who seek antibiotics without a prescription may not be able to purchase these products at pharmacies, this may increase antibiotic demand at convenience stores. We surmise that increased demand at convenience stores will be reflected by increased availability of antibiotics in these establishments assuming that supply chains are responsive to increased demand. Herein, we aimed to establish how antibiotic dispensing regulations in Guatemala impacted the demand for antibiotics at convenience stores by estimating the availability of antibiotics in these establishments.

## Materials and methods

### Study design and setting

We carried out a repeated measures study collecting data before and after antibiotic legislation was passed in Guatemala in August of 2019. Published data from 2016 and 2019 documented the availability of antibiotics in six communities within four municipalities situated in three departments of Guatemala [14]. A second measurement was taken in the same communities to quantify the availability of antibiotics in 2020 and 2021. The selection of convenience stores was based on a previously published study [14]. In brief, areas were defined using Google Earth® maps based on the population density within the borders of each municipality [14]. Subsequently, convenience stores were confirmed on the ground by census and were identified as commercial establishments selling a diversity of products (household cleaning products, personal hygiene products, and basic food products) in relatively small volumes. Stores were classified as participants in the 2016/2019 baseline study, or as new stores identified in 2020/2021. All convenience stores within each community identified in the census were approached for participation, except for stores in Guatemala City. Because of the high density of convenience stores in Guatemala City, a proportionate sample of the population was selected, assuming 50% of stores sell antibiotics, a 5% margin of error, and a 95% confidence interval, and 100% acceptance rate (*n*=169).

After completing the census, and after random selection of stores in Guatemala City, enumerators approached convenience stores to invite them to participate. All convenience store attendants who participated in the study were consented before responding to a brief questionnaire. If stores were closed, they were visited up to three times before being excluded from the study. The questionnaire was previously published [14] and was used to collect information on the type of antibiotic, antibiotic name, manufacturer, dosage and price. Additional questions were added for the 2020/2021 study, including asking the attendant if a prescription was required to purchase antibiotics. Expiration dates and confirmation of the active ingredient on packaging were collected by inspecting medication packaging provided by store attendant. And finally, the attendant was asked if antibiotics were sold for use in animals to explore the potential that human-intended antibiotics might be diverted to veterinary applications. Data collection in the municipality of Antigua was conducted during the last quarter of 2020. In Guatemala City, San Juan Ostuncalco and Coatepeque, the survey was conducted during the first quarter of 2021.

### Ethical approval

This study was reviewed and approved by the Research Ethics Committee of the Center for Health Studies of University del Valle, under Protocol No. 229-02-2021. The committee approved a waiver of signed consent and allowed investigators to carry out oral informed consent for study participation with all study participants.

### Data collection and analysis

Data was collected in electronic format, using a tablet and the REDCap® application. The descriptive and statistical analysis were carried out using OpenEpi version 3.01 and R [15]. A 2-sample *z* test was used to compare two proportions (using software from OpenEpi) to identify differences between municipalities and to compare data before and after antibiotic regulations. We used the McNemar test for paired nominal data with continuity correction to describe the proportion of paired convenience stores selling antibiotics before and after antibiotic regulations. Comparison of marginal frequencies were used to indicate differences in the number of convenience stores that were available before and after antibiotic regulations were in effect. A Bonferroni correction for *P*-values was used after dividing alpha (0.05) by the number of multiple tests.

## Results

During the 2020 and 2021 census, 532 convenience stores were identified of which 91% (484/532) participated in the survey including 172/532 stores (35% of total) in San Juan Ostuncalco, 125/532 stores (26%) in Coatepeque, 129/532 stores (27%) in Guatemala City, and 58/532 stores (12%) in Antigua Guatemala. Of the 484 participating convenience stores, 67% (323/484) sold antibiotics. Approximately half (48%, 157/323) of stores reported having at least one type of antibiotic, 37% (119/323) sold two antibiotics, and 13% (43/323) sold three or more different antibiotics. Amoxicillin was available in 79% (253/322) of the convenience stores, tetracycline capsules were available in 46% (149/323) of the stores, and tetracycline in powder form (tetracycline hydrochloride) was sold in 38% (122/323) of the stores. In San Juan Ostuncalco, six stores sold *furaltamicina* or Furazolidone (nitrofuran antibiotic) in 3-g packets, and in one store trimethoprim/sulfamethoxazole was available as a pediatric suspension. Caplin Point was the most common manufacturer of available amoxicillin capsules (93%, 235/253), Therfarm manufactured most tetracycline capsules (91%, 136/149), and Sante manufactured the tetracycline powder (100%, 122/122). Most stores (98%, 317/323) among the four municipalities indicated they did not require a prescription to sell antibiotics. Expiration dates were not available for blisters/packets/antibiotic packaging for 63% of medications examined (255/402) (Table 1). Nineteen percent (47/253) of the amoxicillin, and 22% (33/149) of the tetracycline was confirmed to be sold for use with people and animals.

**Table 1:** Information gathered from antibiotic packaging found at convenience stores from four municipalities of Guatemala, 2020-2021. Only information from the two most common antibiotics is shown.

**Packaging information**	**Amoxicillin** **n**^a^** = 253**	**Tetracycline** **n**^a^** = 149**	**Total** **n**^a^** = 402**
	**n (%)**	**n (%)**	**n (%)**
Expiration date available	93 (37%)	54 (36%)	147 (37%)
Mass (mg) of antibiotic indicated on package^b^	205 (81%)	105 (70%)	310 (77%)
Active ingredient identifiable on package	219 (87%)	110 (74%)	329 (82%)
Package insert available	17 (7%)	2 (1%)	19 (5%)

^a^Number of stores where indicated antibiotics were available

^b^Mass of drug on the basis of individual units (capsules and tablets only)

### Comparison of data before and after antibiotic regulation

There was no statistical difference in the percentage of convenience stores that sold any antibiotic before and after adoption of dispensing regulations [66.6% (295/443) and 66.7% (323/484), respectively, *P*=0.96], and there was no statistical difference in the number of stores selling amoxicillin before vs. after antibiotic regulation passed [83% (246/443) and 79% (253/484), respectively). The proportion of stores with tetracycline capsules available was 20% lower after antibiotic regulation (*P*<0.01), Table 2.

**Table 2:** Availability of antibiotic capsules in convenience stores before (2016, 2019) and after (2020 and 2021) antibiotic regulation

Municipalities	**Amoxicillin**				**Tetracycline**			
	**2016, 2019**		**2020-21**		**2016, 2019**		**2020-21**	
	N^a^	n^b^ (%)	N	n (%)	N	n (%)	N	n (%)
San Juan Ostuncalco	45	37 (82%)	97	59 (61%)	45	15 (33%)	97	49 (51%)
Coatepeque	58	39 (67%)	88	70 (80%)	58	32 (55%)	88	41 (47%)
Guatemala City	155	142 (92%)	106	102 (97%)	155	128 (83%)	106	47 (44%)*
Antigua	37	28 (76%)	32	22 (69%)	37	20 (54%)	32	12 (38%)
Total:	295	246 (83%)	323	253 (79%)	295	195 (66%)	323	149 (46%)*

^a^N, Number of stores in specified municipality

^b^n, Number of stores where antibiotics were available

^*^P<0.01, *z*-test to compare proportions between 2016 and 2019 versus 2021-2022. A Bonferroni correction was applied assuming alpha = 0.05 divided by five pairwise comparisons

### Comparison of convenience stores enrolled in both study periods

Approximately one-third of stores participated in both phases of the study (36%, 158/443) of which 99% (157/158) consented to participate (Table 3). The majority (57%, 90/158) of stores were from the department of Quetzaltenango (municipalities, San Juan Ostuncalco and Coatepeque), 20% (32/158) were from the department of Sacatepequez (municipality of Antigua), and 17% (26/158) were from the department of Guatemala (municipality, Guatemala City). Most stores sold OTC antibiotics after antibiotic regulations were implemented (66%, 104/157), with 49% (77/157) selling amoxicillin, and 32% (50/157) selling tetracycline. Fifteen percent (24/157) of stores sold antibiotics before 2019 but not in 2020, consistent with regulatory compliance. More stores, however, sold antibiotics after regulations were passed (25%, 39/157).

**Table 3:** Changes in antibiotic availability in convenience stores before and after antibiotic regulation

	**Number of stores with antibiotics available before but not available after regulation**	**Number of stores with antibiotics not available before but available after regulation**
**All stores**	***n*=157 (%)**	***n* =157 (%)**
Any antibiotic	24 (15%)	39 (25%)
Amoxicillin	32 (20%)	33 (21%)
Tetracycline	39 (25%)	23 (15%)
**Quetzaltenango**^a^	***n*=99**	***n*=99**
Any antibiotic	12 (12%)	31 (31%*)
Amoxicillin	16 (16%)	23 (23%)
Tetracycline	24 (24%)	13 (13%*)
**Saquetepequez**^b^	***n*=32**	***n*=32**
Any antibiotic	7 (23%)	4 (13%)
Amoxicillin	9 (28%)	6 (19%)
Tetracycline	8 (25%)	6 (19%)
**Guatemala City**	***n*=26**	***n*=26**
Any antibiotic	5 (19%)	4 (15%)
Amoxicillin	5 (19%)	4 (15%)
Tetracycline	7 (27%)	4 (15%)

^a^Two municipalities in the department of Quetzaltenango were included, San Juan Ostuncalco and Coatepeque

^b^One town, Antigua, in the municipality of Saquetepequez, was included

^*^P<0.017, McNemar test for paired data with continuity correction. A Bonferroni correction was applied assuming alpha = 0.05 divided by three pairwise comparisons for each municipality grouping

## Discussion

Antibiotics remain widely available in convenience stores despite legislation requiring a medical prescription to obtain antibiotics (Ministerial Order 181-2019), which is consistent with ongoing challenges to enforce prescription regulations in Latin America [16, 17]. Herein, we show that antibiotics are continually available in convenience stores in Guatemala without a prescription, often with unknown expiration status, and available for use in humans and animals regardless of the antibiotic regulations established for pharmacies. Convenience stores are not regulated by the Ministry of Health, and they cannot obtain licenses to sell OTC or prescription medications. Nevertheless, it is clear that the licensing requirement is not inhibiting antibiotic vending at these establishments.

New prescription requirements at pharmacies might drive more demand to convenience stores, which could be reflected by more stores selling these products. For stores that were sampled during both time periods, we found an increase in the proportion of stores selling antibiotics during the pandemic phase of the study, but this difference was not statistically significant (15% and 25%, respectively) except for the department of Quetzaltenango, but results were mixed with more stores selling antibiotics overall but fewer stores selling tetracycline specifically (Table 3). Drawing robust inferences from this mixed outcome is not possible without more information about the potential for stockouts, shifting consumer preferences, and trends in illness in the consumer population.

Our findings may have been further confounded by changes in demand due to the SARS-CoV-2 (COVID-19) pandemic [18], which was ongoing during the second phase of this study. The pandemic likely imposed economic pressures on low-income families, increased fear about accessing healthcare facilities, and potentially increased rates of self-medication. Furthermore, it is possible that pharmacies started referring consumers without prescriptions to convenience stores for OTC purchases as was reported in Pakistan [19]. It is also possible that antibiotic demand changed with increasing frequency of pandemic-associated respiratory symptoms as reported elsewhere [20, 21], but we detected no statistically significant increase in use of amoxicillin specifically (Table 3), which is the preferred antibiotic by consumers interested in self-medicating for respiratory symptoms (Ramay et al. unpub. data). Finally, it is possible that stocking practices in some convenience stores were influenced by speculation about potential increased demand for antibiotics during the pandemic rather than reflecting an actual increase in consumer demand at these establishments. All of these factors may have interacted in a manner that negated our ability to detect increases or decreases in demand due to the implementation of a prescription requirement at pharmacies.

One limitation of our study is that we were only able to collect data from four municipalities and these may not be representative of other communities in Guatemala or other communities in Central America. Furthermore, small sample sizes at the municipal level may have reduced our ability to detect statistical differences in proportions of antibiotics available. Additionally, we did not collect information on potential independent factors that may influence antibiotic vending including sociodemographic factors of the customer population, and changes in disease prevalence in surrounding communities. Furthermore, our findings do not reflect or explore effectiveness of regulation in pharmacies, but rather the potential unintended consequences regarding how these regulations impact antibiotic availability in convenience stores outside the current regulatory framework.

## Conclusion

We found that amoxicillin and tetracycline are consistently available in convenience stores located in the Guatemalan municipalities included in this study despite efforts to regulate antibiotic sales through prescription requirements at pharmacies. Antibiotic regulations that are apparently unimplemented or unenforced in dispensaries that fall outside of the jurisdiction of the Ministry of Health represent an ongoing challenge for antibiotic stewardship.

## Data Availability

The dataset(s) supporting the conclusions of this article may be provided upon request to corresponding author.
